# A Comprehensive, Analytical Narrative Review of Polysaccharides from the Red Seaweed *Gracilaria*: Pharmaceutical Applications and Mechanistic Insights for Human Health

**DOI:** 10.3390/nu17050744

**Published:** 2025-02-20

**Authors:** Deepesh Khandwal, Sapna Patel, Abhay Kumar Pandey, Avinash Mishra

**Affiliations:** 1 Division of Applied Phycology and Biotechnology, CSIR-Central Salt and Marine Chemicals Research Institute, Bhavnagar 364002, India; 2 Academy of Scientific and Innovative Research (AcSIR), Ghaziabad 201002, India

**Keywords:** algae, antiproliferative activity, sulphated polysaccharides, functional food, human health, anti-inflammatory, immunomodulation, nutraceutical, seaweed

## Abstract

*Gracilaria* species, a widely distributed genus of red macroalgae, have gathered significant attention for their diverse medical applications attributable to their bioactive sulphated polysaccharides (SPs). This review examines the global narrative of various *Gracilaria* SP applications in terms of their therapeutic potential and mechanistic insights into the use of these SPs against a range of medical conditions, including cancer, inflammation, neurodegenerative disorders, diabetes, and immune dysfunctions. SPs extracted from *G. lemaneiformis* and *G. fisheri* have demonstrated potent anti-tumour activities by inducing apoptosis through various mechanisms, including the upregulation of CD8^+^ T cells and IL-2, inhibition of EGFR/MAPK/ERK signalling pathways, and activation of the Fas/FasL pathway. Selenium nanoparticles (SeNPs) conjugated with SPs further enhanced the targeted delivery and efficacy of these SPs against glioblastoma by the downregulation of ROS followed by the activation of p53, MAPK, and AKT pathways. The anti-inflammatory properties of SPs are evidenced by key suppressive inflammatory markers like NO, TNF-α, IL-1β, and IL-6 in mutant rodent models. SPs from *G. cornea* and *G. birdiae* effectively reduce neutrophil migration and vascular permeability, offering potential treatments for acute inflammation and conditions such as colitis by modulating pathways involving COX-2 and NF-κB. Neuroprotective effects by SPs (from *G. cornea* and *G. gracili*) studied in 6-OHDA-induced rats, which mitigate oxidative stress and enhance neuronal cell viability, facilitate the management of neurodegenerative diseases like Parkinson’s and Alzheimer’s. Regarding the hypoglycaemic effect, SPs from *G. lemaneiformis* exhibit a glucose-modulating response by improving insulin regulation, inhibiting α-amylase activity, repairing pancreatic β-cells, and modulating lipid metabolism. Moreover, immunomodulatory activities of *Gracilaria*-derived SPs include the stimulation of macrophages, T-cell proliferation, and cytokine production, underscoring their potential as functional food and immunotherapeutic agents. Recently, *Gracilaria*-derived SPs have been found to modulate gut microbiota, promote SCFA production, and enhance gut microbials, suggesting their potential as prebiotic agents (*G. rubra* and *G. lemaneiformis*). This review highlights the multifaceted medical applications of *Gracilaria* sulphated polysaccharides, providing detailed mechanistic insights and suggesting avenues for future clinical translation and therapeutic innovations.

## 1. Introduction

The seaweed genus *Gracilaria*, found across tropical and temperate areas, stands as one of the largest genera within the Gracilariaceae family [1]. Intertidal zones and tropical waters contain more than 150,000 kinds of seaweed that are an important primary source for many natural products and medically and commercially relevant derivatives [2]. Globally, there have been around 300 identified species of *Gracilaria*, with 160 of them formally recognized from a taxonomic perspective. Many of these *Gracilaria* spp. have the potential to serve as primary sources of agar [1]. Furthermore, it has been documented that substances found in red, brown, and green algae exhibit a range of advantageous biological properties, including, but not limited to, antioxidative, anti-cancer, antibacterial, antifungal, anti-parasitic, antiviral, and cell-growth-inhibiting actions [3].

Evidence from archaeological sites in Chile suggests that the first use of macroalgae by humans occurred during the Neolithic era, approximately 14,000 years ago. For example, people living in coastal areas have been gathering and harvesting certain types of seaweed for domestic purposes. This practice dates back to ancient times in countries such as Japan (13,000 BC), China (2700 BC), Egypt (1550 BC), and India (300 BC), and these marine plants have been used traditionally as folk medicine to treat a variety of illnesses and diseases. For example, seaweed in the Mediterranean region was used for medicinal and animal feed purposes and as a dye during the ancient Greek and Roman eras. Red algae found in the Mediterranean was used to treat parasitic worms, a practice dating back to the pre-Christian era [4]. However, only wild seaweed was available during this time, limiting its use as a food source up to the Middle Ages. Throughout numerous parts of the globe, particularly in East Asian nations like China, Korea, and Japan, a wide range of seaweed species have been an integral part of both traditional medicine and the food industry, with a history spanning more than six centuries [2].

In recent decades, natural polysaccharides have qualified as unique raw materials due to their characteristics, such as nontoxicity, high compatibility and degradability, hydrophilicity, and protectiveness, which have attracted the attention of pharmaceutical and cosmetic industries. The Japanese discovered agar in the 17th century, and *Gracilaria* spp. were commonly used as a source of agar the [5]. *Gracilaria* species are preferred in China as a food source and a binding material [6]. They are edible and consumed mainly in Japan, Southeast Asia, Hawaii, and the Caribbean. *Gracilaria*-derived agar is a food-grade agar commonly used as a gelling and stabilising agent in the dessert and baking industries. It is instrumental in creating desserts like jelly candies, icing, and pies. Agar has been popular in Western cuisine as a weight loss aid and as a substitute for gelatine derived from animals. It is also used in soups, snacks, and dishes in Japan [7].

Besides this conventional application of galactans, diverse medical and therapeutic approaches have been reported, as *Gracilaria* spp. polysaccharides [8] show anti-cancer [9], anti-inflammatory [10], anti-diabetic [11], immunostimulatory [12], and anti-neurodegenerative [13] activities and help in maintaining gut microbiota homeostasis [14]. This range of bioactivity is influenced by their molecular mass, monosaccharide composition, sulphation level, and chain length, which are influenced by taxonomy, environmental factors (salinity, temperature, light, nutrients), biotic interactions (e.g., epiphytism), and the extraction/purification methodology employed [15]. Thus, optimising these processes is crucial for maximising the sulphation level, purity, and bioactivity, as well as the potential discovery of novel compounds for therapeutic purposes [16].

**Figure 1:**
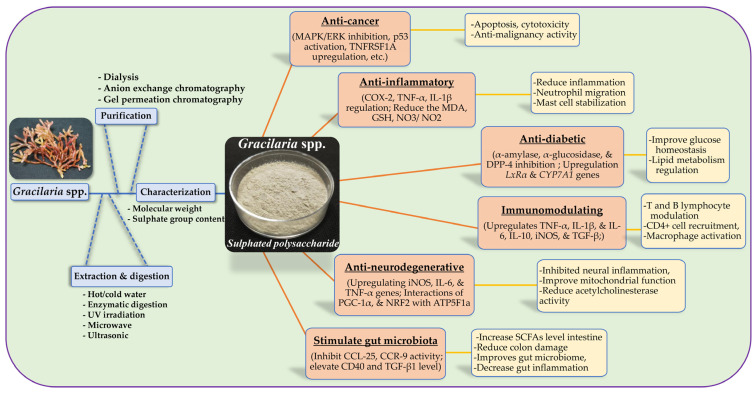
Schematic workflow for sulphated polysaccharides from *Gracilaria* spp.: extraction, purification, characterization and different biological activities including anti-cancer, anti-inflammatory, anti-diabetic, anti-neurodegenerative, immunomodulatory, and gut microbiota modulation.

This review comprehensively examines the diverse therapeutic potential of sulphated polysaccharides (SPs) derived from *Gracilaria* spp. It delves into the underlying mechanisms by which these SPs exert their biological activities across a range of medical applications, including for their anti-cancer, anti-inflammatory, antioxidative, immunomodulatory, and antidiabetic effects, as a neuroprotectant, and as a prebiotic supplement (Figure 1). By synthesising current research, this review aims to provide a deeper understanding of the structure–activity relationships of *Gracilaria* SPs, highlighting key structural features that contribute to their therapeutic efficacy. The review further identifies opportunities for future research, emphasising the importance of preclinical investigations to translate these findings into novel therapies against chronic diseases.

## 2. Effect of Sulfation and Molecular Weight on *Gracilaria* Polysaccharide Bioactivity

Polysaccharides in *Gracilaria* are composed of β-D-galactopyranose and 3,6-anhydro-α-l-galactopyranose units joined by alternating α-(1→3) and β-(1→4) linkages to form polysaccharide chains. These are coiled into a helical secondary structure, giving the agarose matrix its conventional hydrogel properties. A fraction of this polymer consists of agaropectin, a heteropolysaccharide comprising a galactose and sulphate groups, along with other substituents such as pyruvic acid, uronic acid and methyl groups. This chemical diversity contributes to it bioactivity and non-gelling consistency. An especially high degree of sulfation complements agaropectin’s antioxidant and radical-scavenging activity [17,18].

The sulphate content and length of a polysaccharide contribute directly or indirectly to its radical-scavenging capacity and bioactivity (compared in Table 1). In the polysaccharide, these parameters depend on the digestion and extraction protocol employed. Further, during chromatographic separation/purification, sulphated polysaccharide (SP) fragments may change their degree of sulfation and molecular weight (Mw), which may affect their therapeutic properties. In the case of *G. rubra*, hot water extraction followed by DEAE-52 cellulose and Sephadex G-50 column purification yields three fractions of SPs: GRPS-1-1 (1310 kDa), GRPS-2-1 (691 kDa) and GRPS-3-2 (923 kDa), with the sulphate contents of 5.96%, 8.96% and 12.03%, respectively. Of the three fractions, GRPS-3-2, due to its high sulphate content, exhibited the highest immunostimulant and antioxidant activity on RAW264.7 cells [19]. SP eluted from *G. lemaneiformis* by hot water extraction and fractionated with DEAE-52 and Sephadex G-100 produced a similar trend, generating GL-1 (4.9 kDa), GL-2 (52 kDa) and GL-3 (67 kDa), with sulphate proportion of 0.45%, 12.63% and 22.16%. Moreover, GL-3 showed higher activity against influenza virus than the other two [20]. This pattern persisted when *G. lemaneiformis* SP fractions were generated to examine their anti-tumour activity and generate GLP1 (5.5 kDa), GLP2 (85 kDa) and GLP3 (82 kDa), with sulphate level of 0%, 10.8% and 23.2%, respectively, using microwaves at 2450 MHz and ultrasonic 40 kHz extraction (DEAE and Sephadex A-50) [21]. The above studies imply that the method employed for the extraction and downstream processing of SP can change SP fraction characteristics, such as the length and sulfation degree. These findings suggest that extraction and downstream processing techniques critically alter the characteristics of SP fragments, where higher sulphate levels generally correlate with enhanced biological activity within the same fractionated extracts (Table 1).

SPs with molecular weights ranging from 1.0 × 10^2^ to 4.0 × 10^3^ kDa appear to exhibit enhanced bioactivity. SPs below this may not adopt conformations conducive to bioactivity. To obtain SPs of the desired Mw, the extraction/purification technique must be optimised to enhance its antioxidant capacity, efficacy and diffusion properties, thus elevating its bioactivity in curing medical conditions [1]. However, low-Mw SPs of 4.9, 5.5 and 11.6 kDa show moderate bioactivity against influenza virus, anti-cancer activity and anti-inflammatory responses, respectively [20,21,22]. Despite their small size, these SPs exhibit significant bioactivity that should not be overlooked. Thus, the extraction and derivatization of short-chain/low-molecular-weight SPs represents a promising avenue for future research into diverse therapeutic applications [23].

**Figure 2:**
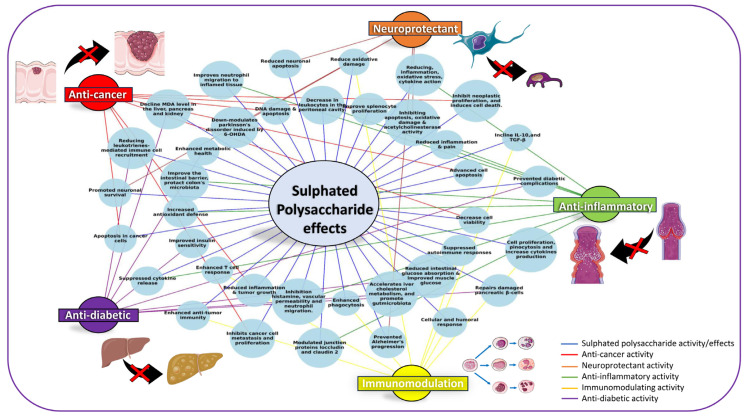
Multidimension approach of *Gracilaria* SP activities for different pharmaceutical and nutraceutical applications.

A wide range of extraction protocols have been adapted to isolate SPs from *Gracilaria* spp. to produce differences in sulfation levels (Table 1). Extraction and treatment with citric acid buffer (pH 2.0) generates 3.06% and 29.82% sulphates [24,25]; 0.3 M NaOH followed by 0.1 M HCl treatment produces a sulphate content of 6.13% [26]; hot water extraction at temperatures ranging from 80 to 100 °C yields 8.14% and 19.64% sulphates [27,28]; and treatment of polysaccharide with enzyme (papain) and UV/H_2_O_2_ yields 15.66% and 33.84% sulphates [10,29]. This wide range of extraction and purification methods provides variation in the degree of SP sulfation ranging from 3.06% to 33.84%. Enzymatic extraction produced SPs with higher sulphate levels (13.22% to 15.66%) than hot water (1.0% to 8.14%) extraction [22,27,29,30]. This highlights the strong correlation between the extraction methodology used and the variation in sulphate levels within SPs of *Gracilaria* spp. Additionally, a higher degree of sulfation implies diverse range of bioactivity, including anti-inflammatory [10] and neuroprotective effects [31], as well as immunomodulatory [19], antidiabetic [1] and apoptotic activities [27]. Extraction techniques play a pivotal role in modulating these properties, providing avenues for tailoring bioactivity through methodical adjustments. These findings underscore the potential of *Gracilaria*-derived polysaccharides as versatile bioactive agents in pharmaceutical and nutraceutical applications (Figure 2).

**Table 1:** Overview on different extraction methods of SPs corresponding to sulphate content, molecular weight (Mw), and bioactivities.

*Gracilaria* spp.	Sulphate Content (%)	Polysaccharide Mw (kDa)	Extraction Technique	Bioactivity	Reference
*G. caudata*	1%	11.6 kDa	Hot water (100 °C, 2h)	Anti-inflammatory	[22]
*G. cornea*	15.66%	-	Papain digestion (60 °C, 6 h)	Anti-inflammatory	[29]
*G. cornea*	26%	-	Acidic extraction (pH 5.0) at 110 °C, 5 h	Neuroprotectant	[31]
*G. fisheri*	8.4%	100 kDa	Hot water (80 °C, 2 h) extraction	Immunostimulatory	[12]
*G. gracilis*	0.1%	-	Hot (100 °C) and cold (25 °C) water extraction	Antidiabetic	[11]
*G. lemaneiformis*	-	<3.5 kDa	Hot water extraction (80 °C, 4 h)	Apoptotic activity	[32]
*G. lemaneiformis*	3.06%	18.5 kDa	Acidic extraction (pH 2.3) at 100 °C, 3 h	Immunostimulatory	[24]
*G. lemaneiformis*	6.13%	13.7 × 10^2^ kDa	0.3 M NaOH and 0.1 M HCl treatment	Anti-tumour activity	[26]
*G. lemaneiformis*	7.08%	<0.5 kDa	Acidic extraction (pH 2.0) at 95 °C, 2 h	Immunostimulatory	[33]
*G. lemaneiformis*	29.82%	21.0 kDa	Citric acid solution (pH 2.0)	Anti-inflammatory	[25]
*G. lemaneiformis*	33.84%	GLP (>700 kDa), 5-GLP (351.76 kDa), 15-GLP (108.37 kDa) and 30-GLP (48.62 kDa)	UV irradiation and H_2_O_2_ treatment	Anti-inflammatory	[10]
*G. lemaneiformis*	9.24% 8.14% 9.16% 8.14%	PGL (123.06 kDa) P-1(14.29 kDa) P-2 (64.78 kDa) P-3 (57.02 kDa)	Hot water extraction (80 °C, 4 h), (DEAE-A25 cellulose and Sephadex G-100)	Anti-proliferative and apoptotic	[27]
*G. lemaneiformis*	19.64%	21.2 kDa	Acidic extraction (pH 2.0) at 100 °C, 2 h	Antidiabetic activity	[28]
*G. lemaneiformis*	22.85%	31.5 kDa	Acidic extraction (pH 2.0) at 100 °C, 2 h	Antidiabetic activity	[34]
*G. lemaneiformis*	8.14% 8.12% 8.24%	GLP (121.89 kDa) GPL1 (57.02 kDa) GLP2 (14.29 kDa)	Hot water extraction 90 °C, 5 h (dialysis)	Antidiabetic, antioxidant	[1]
*G. lemaneiformis*	0.45% 12.63% 22.16%	GL-1 (4.9 kDa) GL-2 (52 kDa) GL-3 (67 kDa)	Hot water extraction (DEAE-52 and Sephadex G-100)	Anti-influenza virus	[20]
*G. lemaneiformis*	0% 10.8% 23.2%	GLP1 (5.5 kDa) GLP2 (85 kDa) GLP3 (82 kDa)	Microwave at 2450 MHz, ultrasonic 40 KHz extraction (DEAE and Sephadex A-50)	Anti-tumour activity	[21]
*G. opuntia*	-	-	Hot water extraction 90 °C, 4 h	Antidiabetic	[35]
*G. rubra*	6.96–12.03%	GRPS-1-1 (1310 kDa), GRPS-2-1 (691 kDa), GRPS-3-2 (923 kDa)	Hot water extraction (DEAE-52 cellulose and Sephadex G-50	Immunostimulatory	[19]

## 3. Mechanism and Effect of *Gracilaria* Sulphated Polysaccharides on Animal Models and Cell Lines in Chronic Disease Management

Bioactive polysaccharides are a subject of intense research across various scientific disciplines due to their diverse structures and abundance in nature. Their therapeutic potency in managing chronic diseases is recognized and cannot be ignored. Here, we focus on the molecular characteristics of *Gracilaria* SPs and explore how their molecular dynamics contribute to their therapeutic effects against chronic diseases.

### 3.1. Anticancer Activity

Cancer is characterized by uncontrolled cell proliferation, disrupting normal body metabolism and leading to tumour formation, organ damage, and systemic dysfunction [36]. Conventional treatments, such as surgery, radiation, chemotherapy, hormone therapy and immunotherapy, are available but often come with significant side effects. Therefore, there is a pressing need for novel anticancer drugs. Previous studies have indicated that seaweeds have the potential to combat and heal carcinomas [37,38].

**Figure 3:**
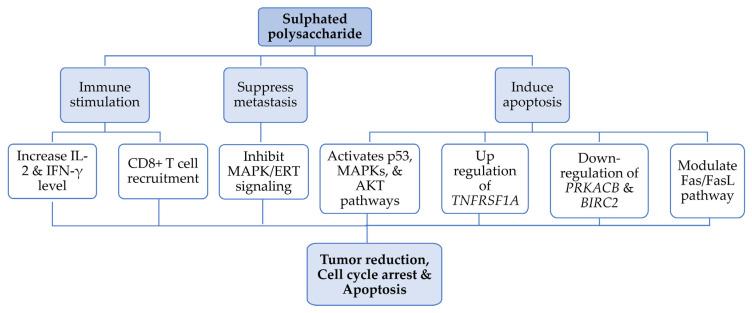
A flowchart representing the molecular mechanism of *Gracilaria* SPs for anti-cancer activity.

Various research studies have demonstrated the molecular mechanism of cancer suppression by SPs extracted from *Gracilaria* spp. (Table 2, Figure 3), such as SP from *G. lemaneiformis*, with an Mw of 1.37 × 10^3^ kDa and a sulphate content of 6.13%, confirmed by HPGPC and GC, respectively. To address its anti-cancer activity, it was administered orally to H22 hepatoma-transplanted female ICR mice. A significant increase in CD8^+^ cells and IL-2 in the peripheral blood of mice with tumours was detected by flow cytometry using CD3^+^ monoclonal antibody. This spike in the cytokinesis level promotes T-cell-like macrophage and splenocyte proliferation and results in tumour reduction and inhibition [26]. Likewise, S180 sarcoma-bearing mice were treated with *Gracilaria* polysaccharide to examine its anti-tumour activity. GLP treatment enhanced IL-2 and IFN-γ in the peripheral blood of neoplastic mice, indicating that polysaccharides from *Gracilaria* enhance immunity in mice [9].

Sae-Lao et al. demonstrated the anti-tumour and anti-malignant activity of *G. fisheri* on human cholangiocarcinoma (HuCCA), a common metastatic disease in Thailand caused by liver fluke infection [38]. They treated HuCCA cells with sulphated agarans from *G. fisheri* and found that *G. fisheri* SP restricted the migration of HuCCA cells via inactivation of matrix metalloproteinases-9 (MMP-9), phosphorylation focal adhesive kinase (FAK) and overexpression of E-cadherin, which restricted cell segregation and migration. Additionally, cell migration could be halted by the inhibition of EGFR and ERK phosphorylation in the EGFR/MAPK/ERK signalling pathway, leading to a reduction in the signal driving migration, survival and invasion. *G. fisheri* SP thus demonstrates potential as a therapeutic agent for the treatment of malignant cancers by effectively targeting pathways involved in cancer cell migration and metastasis [38].

A transcriptomic analysis was performed on the tumour inhibitory effect of *Gracilariopsis lemaneiformis* (order Gracilariales) PS on three different cell lines, MKN45, A549 and HeLa. [32]. Transcriptomic data revealed approximately 758 genes involved directly or indirectly in apoptosis and cell division. mRNA sequencing revealed that an increased level of *TNFRSF1 A* mRNA was associated with apoptosis induction and TNF-α receptor activation. By contrast, *PRKACB* and *BIRC2* levels declined, inhibiting apoptosis in the tumour cell line. In brief, *G. lemaneiformis* PS induces apoptosis and cell cycle arrest in a time- and concentration-dependent manner, making it a candidate drug for lung cancer treatment [32]. Further, Kang et al. in 2017 characterised *G. lemaneiformis* PS to understand its exact apoptotic mechanism based on previously generated transcriptomics data [27]. Using Sephadex G100 gel chromatography, they found 123.06 kDa neutral PS, mainly consisting of three fractions, including 14.29, 64.78 and 57.02 kDa. Using siRNA mediated knocking down of Fas protein; their investigation revealed that PS from *G. lemaneiformis* has anti-proliferative and apoptotic activity correlated to the Fas/FasL signaling pathway, which regulates programmed cell death in the human lung cancer cell line A549. Knockdown of Fas protein inhibits cell proliferation and triggers apoptosis. Thus, Fas is a potential anti-tumour drug target, but additional work is necessary to explore the activity of the Fas/FasL pathway before advancing to clinical trials [27].

The nanobiotechnology approach was employed by Jiang et al. for targeted drug delivery using *G. lemaneiformis* (GLP) docked with SeNP_s,_ (selenium nanoparticles), both individually reported to have anticancer activity [23]. Initially, they found that GLP exhibits a fairly high affinity for *α_v_β_3_* integrin, a receptor for cell adhesion molecules and overexpressed on malignant tumours such as glioma, ovarian cancer and breast cancer. They integrated GLP with SeNP_s_ and studied its anti-glioblastoma properties. In six malignant tumour cell lines (HepG-2, Hela, MCF-7, U87, C6 and A375 cells) and three standard human cell lines (Chem-5, HK-2 and L02), they found that GLP−SeNPs significantly inhibited U87, a cell line with high integrin expression level, with IC_50_ = 9.1 ± 1.53 μM, and C6, a cell line with low integrin expression level, showing an IC_50_ value of 27.6 ± 3.13 μM. By contrast, GLP−SeNPs were not cytotoxic toward normal brain glial cells (Chem-5: IC_50_ = 159.9 ± 9.73 μM) and L02 cells (human liver cells: IC_50_ = 95.6 ± 7.68 μM). They concluded that GLP−SeNPs exhibited significant selectivity in distinguishing between normal and cancerous cells by binding to and interacting with the integrin receptor [23]. This also triggered the downregulation of ROS, followed by activation of the p53, MAPK and AKT pathways, leading to advanced cell apoptosis. In brief, nanomaterial-based targeted nano-drug delivery is a promising strategy for the treatment of glioblastoma and other related tumours [23]. These findings suggest that *Gracilaria* SPs hold therapeutic promise as potent antineoplastic adjuvants in cancer treatment. Further research, including clinical trials, is critical to fully explore SP extraction optimization, efficacy, safety and delivery methods for targeted cancer therapy.

### 3.2. Anti-Inflammatory Activity

**Figure 4:**
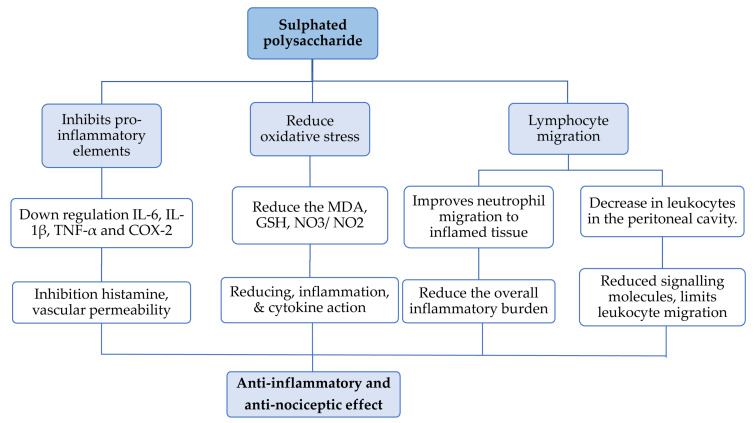
A flowchart representing the molecular mechanism of *Gracilaria* SPs for anti- inflammation activity.

Inflammation, a natural immune response to injury, infection, and stress, can become detrimental if prolonged, contributing to diseases such as asthma, arthritis, neurodegeneration and cancer. While current anti-inflammatory therapies (steroids and non-steroidal drugs) have significant side effects, the need for safer and more effective anti-inflammatory agents is critical [39]. Studies have demonstrated the anti-inflammatory mechanisms of SPs from *Gracilaria* spp. (Table 2, Figure 4). Further, to understand their anti-inflammatory activity, SPs (700 kDa) from *G. lemaneiformis* were treated with UV irradiation (6500 mJ/cm^2^) and H_2_O_2_ (50 mmol/L) for 5, 15 and 30 min. UV/H_2_O_2_ significantly decrease the viscosity, particle size, surface and molecular weight to 351.76, 108.37 and 48.62 kDa for 5, 15 and 30 min, respectively. Among the PSs, 5 min and 351.76 kDa showed the best anti-inflammatory activity by inhibiting nitric oxide, TNF-α and IL-6 by 60.49%, 62.81% and 36.29%, respectively, on the IEC-6 cell line. In brief, PS fractions obtained after UV/H_2_O_2_ treatment were low in Mw and size, which changed their surface morphology without altering their biochemistry. Thus, by inhibiting NO, TNF-α and IL-6 with a low Mw, GLPs can be highly effective in managing inflammatory and autoimmune diseases [10]. Another PS from *G. cornea* with a sulphate content of 15.66% was isolated by papain digestion (60 °C, 6 h) and tested for its ability to inhibit inflammation in carrageenan-induced rat paw oedema. After intraperitoneal administration of GLP in 3, 9 and 27 mg/kg doses, PS reduced neutrophil migration. The 3 and 9 mg/kg doses inhibited oedema in rats, which was confirmed by the myeloperoxidase activity assay. Administration of a higher dose (27 mg/kg) resulted in an observed antinociceptive effect (suppression of pain detection response) in rats, leading to a reduction in latency time. After 14 consecutive days, histopathological evaluations confirmed that PS treatment had no adverse effect on internal organs, supporting the potential use of *G. cornea* PS as an anti-inflammatory and antinociceptive agent [29].

Polysaccharide extract from *G. lemaneiformis* with an Mw of 21.0 kDa and a sulphate content of 29.82% was tested on colitis (intestinal inflammation) induced by dextran sulphate sodium (DSS) in Balb/c mice [25]. The study demonstrated that the sulphate group is a sulphated polysaccharide that contains (1→3)-linked glycosidic bonds, which contribute to its cytokine-suppressing effects. Animal experiments showed that SP can prevent colitis by reducing inflammatory responses, enhancing intestinal barrier function and forming a defensive layer on the surface of intestinal epithelial cells. These changes help to maintain the complete colon microstructure and immune system suppression associated with colitis, by reducing the contents of endotoxin and lipopolysaccharide-binding protein produced by bacteria. Additionally, SPs restrain IFN-γ, IL-6 and IL-1β in the colon. The study suggests that SP has the potential to maintain intestinal health by preserving colon structure and can be considered a potential candidate for treating intestinal inflammation [25].

*G. cornea* SP was isolated to understand and validate inflammation-inhibitory mechanisms acting on paw oedema induced by compound 48/80, a synthetic polymer used to study allergic reactions, inflammation and mast cell biology, in rats [29]. SP was subcutaneously introduced (3, 9 or 27 mg/kg), and the 9 mg/kg dose reduced rat paw oedema induced by compound 48/80. PS also inhibited oedema in animals with intact mast cells but not in those with degranulated mast cells, suggesting a protective effect on mast cell membranes. Thus, SP from *G. cornea* exhibits anti-inflammatory properties by reducing histamine, vascular permeability and neutrophil migration, all of which are factors in the acute inflammatory process. SP activity down-modulates COX-2, TNFα and IL-1β, confirmed by qRT-PCR and immunohistochemistry analyses. Thus, SP from *G. cornea* can act on several different pathways involved in the inflammatory process [29]

Likewise, SP (11.6 kDa) from *G. caudata* was administered at 1, 3 and 10 mg/kg intraperitoneally to determine the dose of PSs required to elicit a cytokine-suppressing effect in mice with ulcerative colitis [22]. The 10 mg/kg dose significantly (*p* < 0.05) reduced the macroscopic characteristics of colitis. SP from *G. caudata* reduces GSH (glutathione) consumption and the levels of MDA, NO_3_/NO_2_ and pro-inflammatory cytokines (IL-1β and TNF-α) while also decreasing iNOS expression. Thus, *G. caudata* SP demonstrates therapeutic potential and can be considered an alternative for the treatment of inflammatory bowel diseases such as ulcerative colitis [22]. de Sousa Oliveira Vanderlei et al. performed similar experiments on *G. birdiae* SP [30]. They introduced SP peritoneally in a carrageenan-induced paw oedema model using Wistar rats. To check SPs for immunomodulatory activity, they administered 10 mg/kg SP and observed that total leukocyte migration was reduced by 52%. This suppression of mononuclear cell infiltration was supported by histopathological evidence [30].

Makkar et al. performed an in vitro study of SPs from *G. opuntia* to investigate their COX/LOX-inhibiting activity [35]. They targeted inflammatory elements such as COX-1, COX-2 and 5-LOX pro-inflammatory enzymes and found lower IC_50_ values (0.01, 0.03 and 0.24 mg/mL, respectively) for the SP than for the positive control aspirin. Inhibiting COX-1, COX-2 and 5-LOX targets key inflammatory mediators, reducing prostaglandin and leukotriene-mediated immune cell recruitment. Combined inhibition provides a balanced anti-inflammatory response, addressing both pathways while minimising complement system activation and inflammatory side effects. So, *G. opuntia* SP has the clinical potential to suppress inflammation-induced conditions such as hyperglycaemia [35]. In 1996, Yoshizawa et al. isolated the polysaccharide fraction from *G. verrucose* and examined it on C57BL/6N mice (ideal for studying immune responses, inflammation and autoimmune diseases) [40]. After 15 days of intraperitoneal administration, they observed that the PSs have macrophage-stimulating activity (dose: 4.0 mg/animal) by stimulating peritoneal exudate cells (PECs) and increasing their phagocytic activity [40]. Based on these findings, we conclude that polysaccharides likely interact with macrophage receptors, such as Toll-like or C-type lectin receptors, activating signalling pathways that enhance cytokine production and phagocytosis. These findings suggest that *G. verrucosa* polysaccharides have potential as immunomodulatory agents to boost innate immunity. These outcomes suggest that *Gracilaria* SPs are promising candidates for developing novel anti-inflammatory therapies. Further research, including clinical trials, to explore their therapeutic potential in managing inflammatory diseases is warranted.

### 3.3. Anti-Diabetic Activity

**Figure 5:**
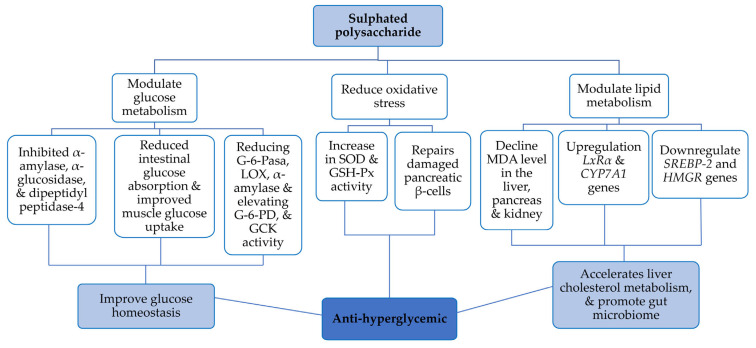
A flowchart representing the molecular mechanism of *Gracilaria* SPs for anti-hyperglycaemic effect.

Diabetes, a metabolic disorder characterized by β-cell destruction or insuline resistance, is classified as type 1 or type 2, respectively. Potential comorbid conditions include cardiovascular disease, obesity, vascular complications, hypertension and gestational diabetes; these factors lead, either directly or indirectly, to organ damage and dysfunction [14,41]. In recent years, *Gracilaria*-derived SPs have garnered significant attention for their potential role in managing diabetes—a growing global health concern, especially among younger populations [14]. Research has revealed diverse molecular mechanisms through which SPs from *Gracilaria* spp. can aid in managing diabetes (Table 2, Figure 5). In line with the above, *Gracilaria* SPs exhibit antidiabetic properties by improving insulin sensitivity and modulating glucose metabolism, drawing attention to its therapeutic potential in diabetes [11,28]. Thus, the hypoglycaemic activity of *G. lemaneiformis* SP, with an Mw of 121.89 kDa (57.02 and 14.29 kDa fractions) and 8.24% sulphate ester, was validated by intraperitoneal administion to alloxan-induced diabetic mice (alloxan induces diabetes mellitus via apoptosis and necrosis of β-cells). Among the isolated SPs, the 57.02 kDa fraction displayed better results than the 14.29 kDa one. After 21 days of treatment, an elevation was observed in the malondialdehyde (MDA) levels in the liver, pancreas and kidneys of diabetic mice. A significant increase in superoxide dismutase (SOD) and glutathione peroxidase (GSH-Px) activity was obsereved in the liver and kidney compared to mice treated with the positive control Glibenclamide (a type-2 diabetes drug). In conclusion, the effects of the 57.02 kDa PS are likely mediated by its antioxidant properties. By reducing oxidative stress and protecting tissues like the pancreas, it supports insulin production and glucose regulation, making it a potential treatment for hyperglycaemia in diabetic patients [1].

Wen et al. isolated a 21.2 kDa low-molecular-weight polysaccharide with a high galactose content and a 19.64% sulphate level from *G. lemaneiformis* [28]. SP was injected into streptozotocin-induced diabetic mice (streptozotocin causes irreversible damage to pancreatic β-cells); after a six-week animal trial, it was found that SP repaired pancreatic β-cells, inhibited α-amylase activity and downregulated lipoxygenase activity. On the other hand, *G. lemaneiformis* PS regulated insulin and blood urea better than metformin (a commercial anti-diabetic drug). Additionally, biochemical assays on diabetic mouse livers revealed elevations in glycolytic enzymes, such as GCK (glucokinase) and G-6-PD (glucose-6-phosphate dehydrogenase), and depression in G-6-Pase (glucose-6-phosphatase) levels. The above synchronised enzymatic mechanisms can increase glucose uptake and decrease hepatic glucose breakdown, improving glucose homeostasis in diabetes. Evidently, low-molecular-weight PS from *G. lemaneiformis* can be an alternative/adjuvant anti-diabetic supplement [28].

In another study, hyperlipidemic mice were supplemented with *G. lemaneiformis* SP (31.5 kDa) to understand its effect on lipid metabolism and the transcription profile [34]. After 40 days of treatment, it was observed that total cholesterol (TC), triglyceride (TG) and free fatty acid levels (FFA) were low in serum compared to positive control (atorvastatin) mice. Under the action of *G. lemaneiformis* SP, *LxRα* and *CYP7A1* gene expression was upregulated, while *SREBP-2* and *HMGR* gene expression was downregulated, accelerating liver cholesterol metabolism and promoting the conversion of primary bile acids to secondary bile acids. PS also promoted the growth of gut microbes such as *Bacteroides*, *364 Ruminococcus_1* and *Lactobacillus*, which participate in bile acid metabolism confirmed by 16s RNA sequencing. People with high cholesterol are prone to cardiovascular diseases, so *G. lemaneiformis* is a potential candidate to lower cholesterol levels in the body [34]. Makkar et al. isolated and studied the SPs from *G. opuntia* to investigate its anti-diabetic properties [35]. They checked the in vitro activity of enzymes involved in carbohydrate breakdown and obtained the following results: α-amylase (IC_50_ 0.04 mg/mL), α-glucosidase (IC_50_ 0.09 mg/mL), and dipeptidyl peptidase-4 (IC_50_ 0.09 mg/mL). The results positively correlate with the anti-diabetic activity of sulphated polysaccharides and prove its therapeutic potential [35].

*G. gracilis* SP were extracted using hot and cold water, then purified by DEAE column chromoatography [11]. These extracts were subsequently tested for enzyme inhibition activity related to type 2 diabetes. An in vito study revealed that both hot and cold water extracted SPs inhibited α-glucosidase and pancreatic lipase, with the IC_50_ ranging from 0.03 to 0.17 mg/mL. After administering a high concentration of SP (1 mg/mL) to male Sprague-Dawley rats, Pillay et al. found that it reduced intestinal glucose absorption and improved muscle glucose uptake more effectively than the standard drug metformin [11]. Additionaly, crude and purified SPs inhibited α-amylase, suggesting that extracts from *G. gracilis* may be useful in the management of type 2 diabetes. These findings suggest that *G. gracilis* SPs may regulate carbohydrate digestion, reduce sugar release in the gut and enhance glucose utilization in peripheral tissues, highlighting their potential as antidiabetic food supplements [11].

The above study demonstrates the various mechanisms by which SPs can mitigate the diabetic condition, including improving insulin sensitivity, modulating glucose metabolism [11,28], enhancing antioxidant activity [1], protecting pancreatic β-cells [28], regulating lipid metabolism [34] and inhibiting carbohydrate-hydrolyzing enzymes [11,35]. Evidently, *Gracilaria* SPs demonstrate potential as novel therapeutic or adjunct agents for managing diabetes and related metabolic disorders.

### 3.4. Immunomodulating Activity

**Figure 6:**
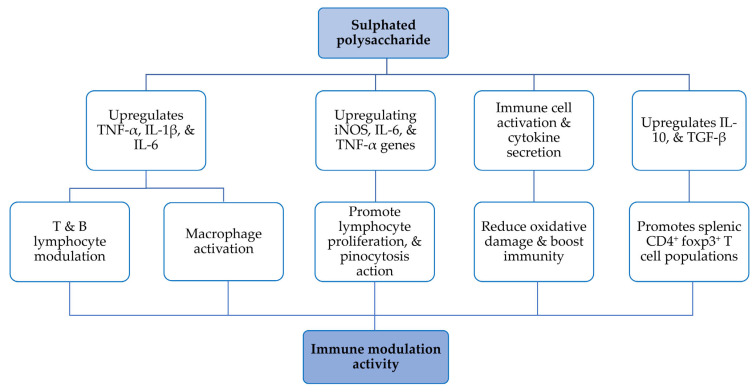
A flowchart representing the molecular mechanism of *Gracilaria* SPs for immunomodulation activity.

Immunomodulation aims to restore cytokine homeostasis, either by suppressing inflammation and regulating immune responses or by boosting a deficient immune system. Cytokines are critical regulators of NK cell activation, maturation, proliferation, cytotoxicity and chemotaxis [42]. Studies have demonstrated the immunomodulatory effects and underlying molecular mechanisms of SPs from *Gracilaria* spp. (Table 2, Figure 6). Thus, hot water extraction of *G. rubra* followed by DEAE-52 cellulose and Sephadex G-50 column chromatography yielded three fractions of SPs: GRPS-1-1, GRPS-2-1 and GRPS-3-2, with molecular weights of 1310, 691 and 923 kDa, respectively [19]. In fact, biochemical tests of all the fractions generated strong antioxidant and radical-scavenging activity. But GRPS-3-2 prevented oxidative damage caused by H_2_O_2_ on PC12 cells and produced stronger immune boosting activity on RAW264.7 cells than the other two fractions of PS. This suggests that *G. rubra* SPs can be introduced as a functional food to induce the immune system [19]. Similarly, hot water extraction of SPs from *G. fisheri* produced two fractions (SG-1 and SG-2), which were validated for their ability to elicit an immune response in murine J774A.1 macrophages [12]. SG-1 and SG-2 have similar sulphate content, around 22 mg/g. Since SG-1 has a higher galactose content, it was chosen for its immunostimulatory activity on macrophages. Administering SP to J774A.1 macrophages revealed an elevation of NO and iNOS levels and also led to a spike in cytokines such as TNF-α, IL-1β and IL-6. In addition, in murine macrophages, SG-1 enhanced phagocytosis and increased the expression and activation of pro-inflammatory elements [12].

Sulphated and acetylated derivatives of PS were extracted from *G. lemaneiformis* to characterise its immunological activity. Single-cell suspensions of B and T lymphocyte from BALB/C mice were proliferated in vitro to test SPs for immunological activity [33]. Three samples, crude PS, sulphated PS and acetylated PS, were used to examine T- and B-cell proliferation. At a dose of 0.064 μg/mL, crude PS exhibited the strongest T-lymphocyte stimulating activity compared to sulphated PS and acetylated PS. Meanwhile, sulphated PS reduced B lymphocyte proliferation, indicating potential use in treating immunosuppressive diseases such as systemic lupus erythematosus, dermatomyositis and scleroderma. This study found that sulphated and acetylated PS from *G. lemaneiformis* significantly enhanced T lymphocyte proliferation, suggesting it as a immunoenhancing supplement [33].

Using a DEAE-Sepharose ion exchange column, SPs extracted from *G. lemaneiformis* by hot water were separated into five fractions—GLP-1, GLP-2, GLP-3, GLP-4, and GLP-5— and checked for immunostimulant activity [24]. Among the fractions, GLP-2 (18.50 kDa) demonstrated the strongest immunostimulant activity. RAW264.7 cells were treated with GLP-2, leading to upregulation of iNOS, IL-6 and TNF-α gene expression; significant enhancement of proliferation and pinocytosis; and the production of oxidative stress and cytokines, such as ROS, NO, IL-6 and TNF-α in RAW264.7 cells. This highlights its potential as an immunomodulatory agent for functional foods and dietary supplements [24].

The studies cited above demonstrate the immunomodulatory potential of *Gracilaria* spp. polysaccharides (SPs), showcasing diverse effects on immune cells. Studies reveal that *Gracilaria* SPs can enhance antioxidant activity, protect against oxidative damage [19], stimulate macrophage activity and cytokine production [12,24] and selectively modulate T and B lymphocyte proliferation [33]. These findings suggest that *Gracilaria* SPs hold promise as immunomodulatory agents for functional foods, dietary supplements and potential therapeutic applications in immune-deficient conditions [43,44].

### 3.5. Anti-Neurodegenerative Activity

**Figure 7:**
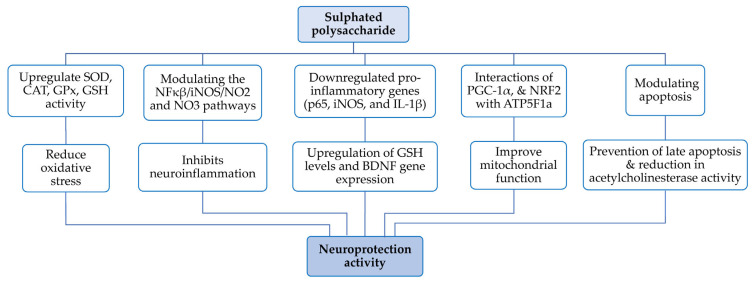
A flowchart representing the molecular mechanism of *Gracilaria* SPs for neuroprotectant activity.

Neurodegenerative diseases, including Alzheimer’s, Parkinson’s, amyotrophic lateral sclerosis, Huntington’s, multiple sclerosis, cerebral ischemia and traumatic brain injury are characterised by age-related, chronic and progressive neuronal loss. Primary contributing factors include oxidative stress, protein misfolding, mitochondrial dysfunction, apoptosis, impaired proteostasis and neuroinflammation [14]. Studies have explored the mechanisms and neuroprotective effects of SPs from *Gracilaria* spp. in the context of neurodegenerative conditions (Table 2, Figure 7). Therefore, SP from *G. cornea* was obtained for testing on neurodegenerative disorders [31]. First, SP was introduced into a PD (Parkinson’s disease) induced rat model (induced by 6-hydroxydopamine, a synthetic neurotoxin) and its effect was observed by removing rat brain segments on the 14th day. The study demonstrated that SP exhibited anti-parkinsonian effects by modulating the NFκβ/iNOS/NO_2_ and NO_3_ pathways, as well as downstream inflammatory and oxidative cascades that damage dopaminergic neurons. SP also downregulated pro-inflammatory genes (p65, iNOS and IL-1β) to basal levels and upregulated the brain-derived neurotrophic factor (BDNF) gene. Thus, it shows neuroprotective effects, likely through upregulation of GSH levels and BDNF gene transcription. These findings suggest SP potential against 6-hydroxydopamine-induced lesions and neurodegenerative disorders [31].

Similarly, hot-water-extracted SP from *G. gracilis* was introduced into the HT-22 cell line (mouse hippocampal cell line, first damaged by 50 µM ZnSO_4_), which induced neurotoxicity and caused a 50% reduction in cell viability [13]. Then, treatment with SP restored cell viability to 95%, preventing late apoptosis and necrosis triggered by zinc, thus revealing the neuroprotective capacity of SPs. Regarding oxidative parameters, SOD, CAT (catalase) and GSH levels were reduced in Zn-induced cells. Adding to this, SP infusion into Zn-impaired HT-22 cells increased antioxidant enzymes and glutathione levels while reducing acetylcholinesterase activity. This supports the notion that SP from *G gracilis* is a potential treatment for neurodegeneration, cholinergic dysfunction and neuroinflammation [13].

Regarding the neuroprotective or neurodegenerative roles of SPs from *Gracilaria*, no sufficient data have been reported. In a review by Meinita et al., the neuroprotective mechanisms of polysaccharides from six different seaweed species (*T. decurrens*, *G. cornea*, *L. japonica*, *C. pyrenoidosa*, *S. platensis* and *F. vesiculosus*) were studied [14]. The review emphasized that the neuroprotective effects of these polysaccharides target multiple biological mechanisms. These include direct modulation of reactive oxygen species (ROS), enhancement of glutathione (GSH) levels and reduction in Nox1 expression. Additionally, these polysaccharides can upregulate critical proteins such as PGC-1α and NRF2, improve mitochondrial respiratory function through interactions with ATP5F1a and increase the activity of antioxidant enzymes, including GPx (glutathione peroxidase), SOD and CAT. Moreover, they modulate the transcription of pathways related to NFκB/iNOS/NO_2_ and NO_3_, influence the immune response via the brain–gut axis and further enhance the activity of antioxidant enzymes like GPx and SOD [14]. Obtaining such promising results from studying various seaweed polysaccharides, it is clear that they hold great potential as neuroprotective agents. Future research should focus on investigating the precise mechanisms of action and the neurodegenerative roles of SPs from *Gracilaria*, as well as evaluating their therapeutic efficacy in preclinical and clinical stages. With continued exploration, these *Gracilaria* SPs could pave the way for novel treatments targeting neurodegenerative conditions.

## 4. Effect on the Gut Microbiota

Humans lack the essential enzymes to directly digest most non-starch polysaccharides. Eventually, these undigested polysaccharides reach the distal intestine where the gut microbiota, possessing carbohydrate-active enzymes (CAZymes), ferment them into short-chain fatty acids (SCFAs). These SCFAs exert beneficial physiological effects on human health. Research confirms that various polysaccharides modulate gut microbiota composition by increasing beneficial microbes and decreasing harmful ones. This beneficial gut microbiome positively affects obesity, type 2 diabetes and metabolic syndrome [45,46].

Table 2 addresses the effect of SPs and oligosaccharides on the mode of action, gut microbe health and effect on the health of model organisms. SPs have shown effective results on gut microbes, increasing and diversifying the number of microbes found in the gut. Studies show that *Gracilaria* polysaccharides favour the gut microbial composition, resulting in a reduction in the Firmicutes/Bacteroidetes (F/B) ratio (biomarker of gut microbiota imbalance) [33,47]. Adding to this, SPs also decrease the abundance of Proteobacteria, a phylum associated with inflammation [47,48]. Furthermore, *Gracilaria* SPs enhance short-chain fatty acid (SCFA) production, particularly acetate, propionate and butyrate, in both in vitro and in vivo models [47,49,50]. Eventually, increases in SCFAs promote the growth of beneficial bacteria like *Roseburia, Faecalibacterium, Alloprevotella, Lactobacillus, Prevotella* and *Ruminococcus* [47,50,51], contributing to improved gut barrier function and overall gut health. These findings highlight the potential of *Gracilaria* spp. and their derived SPs as functional food ingredients and dietary supplements for promoting gut health and mitigating gut-related disorders.

**Table 2:** Effects of *Gracilaria* SPs on different test models, highlighting their mode of action and potential therapeutic applications.

*Gracilaria* spp.	Test Models/Cell Lines	Pathway/Mechanism	Effect on Test Models/Cell Lines	Author’s Conclusions	References
*Anti-cancer activity*					
*G. dominguensis*	Effect on Ehrlich ascites carcinoma (EAC) in mice	-	Anti-malignant, induced apoptosis and anti-tumour activity	A complementary drug in case of cancer	[52]
*G. fisheri*	(Cholangiocarcinoma) CCA cells (HuCCA-1 & RMCCA-1)	Inhibition of MAPK/ERK signalling transduction pathway	Inhibits cancer cell metastasis and proliferation	Therapeutic potential on CCA treatment	[38]
*G. foliifera*	MDA-MB-231, MCF-7 and T-47D	-	Enhanced cytotoxicity and suppressed cell proliferation	Cytotoxic effect	[37]
*G. lemaneiformis* (GLP−SeNPs)	U87 & C6 like glioma cell lines	Activation of p53, MAPKs, and AKT pathways by binding with *α_v_β_3_* integrin	Advanced cell apoptosis	Anti-glioblastoma activity	[23]
*G. lemaneiformis*	Transplant mice with H22 hepatoma cell	Increase IL-2 and CD8^+^ T cells in tumour transplant mice	Improved splenocyte proliferation results in tumour suppression	Clinical supplement	[26]
*G. lemaneiformis*	MKN45, A549 and HeLa	Upregulation of *TNFRSF1A* and downregulation of *PRKACB* and *BIRC2* mRNA level	Induce apoptosis, decrease cell viability and cell cycle arrest	Anti-lung cancer activity	[32]
*G. lemaneiformis*	A549, MKN28 and B16	Induces apoptosis by Fas/FasL pathway in cell line	Inhibits neoplastic proliferation, and induces cell death	Novel drug against neoplastic diseases	[27]
*Gracilaria* spp.	S 180 sarcoma-affected mice,	Inflated level of IL-2 and IFN-γ	Improve immunity of affected mice	Anti-tumour activity	[47]
***Anti-inflammatory activity***					
*G. birdiae*	Male Swiss mice or Wistar rats. Induced by carrageenan and dextron	Leukocyte migration	Decrease in leukocytes in the peritoneal cavity	Anti-inflammatory effects	[30]
*G. caudata*	Dexamethasone induced male swiss mice	Reduce the myeloperoxidase (MPO) activity, glutathione (GSH) consumption, malondialdehyde (MDA), NO_3_/ NO_2_, pro-inflammatory cytokine concentrations and reduction in iNOS expression	Reducing inflammatory infiltrate, oxidative stress, pro-inflammatory cytokine action and iNOS expression	Bowel anti-inflammatory activity	[22]
*G. caudate*	Male Swiss mice	Reduction in joint oedema, MPO activity cell influx, IL-1β and NO levels	Improves neutrophil migration to inflamed tissue, inhibits hyper-nociception, oedema	Treatment for arthritic inflammation	[53]
*G. cornea*	Wistar rats (induced inflammation model)	Anti-inflammatory action mast cell stabilization and IL-1β, TNF-α and COX-2 downregulation	Inhibition of histamine, vascular permeability and neutrophil migration	Anti-inflammatory effects	[29]
*G. fisheri*	ICR/Mlac male mice (acetic acid-induced colitis model)	Reduced TNF-α, IL 1β and modulated tight junction proteins (occludin and claudin 2)	Anti-inflammatory effects, modulation of colonic epithelial barrier dysfunction	Anti-inflammatory, colonic epithelial barrier dysfunction	[50]
*G. lemaneiformis*	Balb/c Mice with colitis induced by dextran sulfate sodium (DSS)	Suppression of TNF-α, IL-1β and IL-6 in colon tissue. Increase myeloperoxidase activity in serum	Improve the intestinal barrier, safeguard the structure of the colon’s microbiota	Prevent intestinal inflammation	[25]
*G. lemaneiformis*	IEC-6 cells	Inhibition of LPS-induced NO, TNF-α and IL-6 production in IEC-6 cells	S-PS fractions possess anti-inflammatory activity	Modulates inflammation and auto-immune diseases	[10]
***Anti-diabetic activity***					
*G. gracilis*	Male Sprague-Dawley rats	Inhibiting gluconeogenesis and promoting glucose utilization in peripheral tissue	Reduced intestinal glucose absorption and improved muscle glucose	Diabetic food supplement	[11]
*G. lemaneiformis*	Alloxan-induced diabetic mice	Significant increase in SOD and GSH-Px antioxidants	Diabetic mice show steep decline in malondialdehyde (MDA) in the liver, pancreas and kidney	Anti-hyperglycemic potent molecule for diabetes	[1]
*G. lemaneiformis*	Streptozotocin-induced diabetic mice	Reducing G-6-Pase and elevating G-6-PD and GCK levels	Repairs the damaged pancreatic β-cells, inhibits α-amylase activity, and downregulates lipoxygenase activity	Anti-diabetic supplements	[28]
*G. lemaneiformis*	Hyper-lipidemic mice	Upregulation of genes *LxRα* and *CYP7A1*	Acceleration of liver cholesterol metabolism, and promote micro flora	Function food for lower cholesterol level	[34]
*G. opuntial*	In vitro antidiabetic activity	Inhibited α-amylase,α-glucosidase and dipeptidyl peptidase-4	Reducing prostaglandin- and leukotriene-mediated immune cell recruitment	Diabetic supplement	[35]
***Immunomodulating activity***					
*G. fisheri*	J774A.1 murine macrophage cell line	Increased the expression of TNF-α, IL-1β and IL-6	Enhanced phagocytosis, increased pro-inflammatory elements	Immunomodulating activity	[12]
*G. lemaneiformis*	T and B lymphocyte from BALB/C mice	Increased the proliferation activity of the T and B lymphocyte	-	Cellular and humoral response	[33]
*G. lemaneiformis*	RAW264.7 cells	Upregulating iNOS, IL-6 and TNF-α gene	Proliferation, pinocytosis and increased cytokine production	Potent immunomodulatory activity	[24]
*G. lemaneiformis*	Ovalbumin (OVA)-induced mouse	Upregulating splenic CD4^+^ foxp3^+^ T cell populations	Increase in serum interleukin-10, transforming growth factor-β	Anti allergic activity	[54]
*G. rubra*	RAW 264.7 and PC12 cells	Immune cell activation and cytokine secretion	Reduce oxidative damage and boost immunity	Immunomodulating activity	[19]
***Neuroprotectant activity***					
*G. cornea*	6-OHDA (6-hydroxydopamine) in rats	Reduce oxidative/nitro active stress and affect monoamine content (In vitro)	Rat model PD induced by 6-OHDA then supplemented with SA	Potential candidate for Parkinson’s disease	[31]
*G. gracilis*	HT-22 cell line (Zn-induced damage)	Upregulation of antioxidant enzymes; SP prevents Zn-induced damages	Inhibiting apoptosis, oxidative damage and acetylcholinesterase activity in Zn necrotic HT-22 cell line	Neuroprotectant in case of Alzheimer’s disease	[13]
***Gut microbiota stimulator***					
*G. fisheri*	ICR/Mlac mice (male) (acetic acid-induced colitis model)	Prevent gastrointestinal dysmotility, potentially by modulating gut microbiota composition and increasing short-chain fatty acid (SCFA) production	Reduced colonic shortening, body weight loss, and disease activity index, and promotes gut smooth muscle contraction	Prebiotic effects	[51]
*G. lemaneiformis*	In vitro digestion and fermentation by human fecal microbiota	-	Modulate and promote the composition of gut microbiome including Proteobacteria, Firmicutes, *Desulfovibrio* and *Bacteroides*	Potential probiotic supplement	[55]
*G. lemaneiformis*	Sixty Duroc × (Landrace × Yorkshire) castrated male pigs	Decrease in hazardous bacteria (Shigella), but increase in beneficial bacteria *Clostridium* and *Lactobacillus*	Elevation in anti-inflammatory and antioxidant compounds (methyl cinnamate, protopanaxatriol, and isovanillic acid)	Functional feed additive	[14]
*G. lemaneiformis*	BALB/c mice on high-fat diet	Gut microbiota changes affect lipid gene expression (via SCFAs)	Reduced hepatic and adipose fat accumulation, improve metabolic syndrome and intestinal diseases.	Food supplement	[49]
*G. lemaneiformis*	Kunming Male mice (dextran sulfate sodium-induced mode)	CCL-25 and CCR-9 level were inhibited, and CD40 and TGF-β1 were increased. Increase in Bacteroidetes, and decrease of Firmicute	Reducing colonic damage, increasing intestinal SCFAs. Increasing inflammation	Functional food for ulcerative colitis patients	[47]
*G. rubra*	In vitro digestion and fermentation by human fecal microbiota	-	Lowered pH, elevated SCFA concentrations, altered gut microbiota composition, and promoted bacterial growth	Potential probiotic	[56]

## 5. Discussion

Managing prolonged health conditions have been a major concern in the scientific community. This review describes the bioactive mechanism of *Gracilaria* SPs in controlling chronic diseases. The anti-cancer activity of SPs exhibited various effects, including decreased cell viability [32], cytotoxicity [37], apoptosis induction [38] and inhibition of tumour cell malignancy [27]. These effects were mediated by various mechanisms, including inhibition of MAPK/ERK signalling in HuCCA-1 cells; activation of p53, MAPKs and AKT pathways in U87 and C6 cell lines; and modulation of *TNFRSF1A* and *PRKACB* expression in MKN45, A549 and HeLa cell lines [23,32,38]. In studies on animal models (tumour mice), SPs induced immune responses, such as improving splenocyte proliferation by increasing IL-2, IFN-γ and CD8^+^ T cells [26,47]. SPs from various *Gracilaria* spp., including *G. birdiae*, demonstrated anti-inflammatory effects in male Swiss mice and Wistar rats by reducing leukocyte migration and decreasing leukocyte counts in the peritoneal cavity [30]. Similarly, *G. caudata* in dexamethasone-induced male Swiss mice reduced MPO activity, oxidative stress markers, and pro-inflammatory cytokine concentrations, alongside downregulation of iNOS expression [22]. In arthritic inflammation models, *G. caudata* reduced joint oedema, MPO activity, and IL-1β and NO levels, improving neutrophil migration and inhibiting hypernociception [53]. *G. cornea* SPs in Wistar rats stabilize mast cells, downregulating IL-1β, TNF-α and COX-2, and inhibiting histamine release, vascular permeability and neutrophil migration [29]. In acetic acid-induced colitis models, *G. fisheri* reduced TNF-α and IL-1β levels while modulating tight junction proteins [50]. *G. lemaneiformis* suppressed TNF-α, IL-1β and IL-6 levels in dextran sulphate sodium (DSS)-induced colitis in Balb/c mice and IEC-6 cells, improving intestinal barrier function and microbiota structure, thus preventing intestinal inflammation [10,25].

Regarding anti-diabetic activity, SPs from *G. gracilis* inhibited gluconeogenesis and promoted glucose utilization in peripheral tissues of male Sprague-Dawley rats, reducing intestinal glucose absorption and improving muscle glucose uptake [11]. In alloxan-induced diabetic mice, *G. lemaneiformis* significantly increased SOD and GSH-Px while reducing MDA levels in the liver, pancreas, and kidney, indicating its anti-hyperglycaemic potential [1]. Similarly, in streptozotocin-induced diabetic mice, *G. lemaneiformis* repaired damaged pancreatic β-cells, inhibited α-amylase activity, and downregulated lipoxygenase activity by modulating G-6-Pase, G-6-PD and GCK levels [28]. In hyperlipidaemic mice, *G. lemaneiformis* upregulated *LxRα* and *CYP7A1* genes, accelerating liver cholesterol metabolism and promoting gut microbiota balance [34]. SPs from *G. fisheri* enhanced immunomodulatory activity in J774A.1 murine macrophage cells by increasing TNF-α, IL-1β and IL-6 expression and promoting phagocytosis [12]. In BALB/c mice, *G. lemaneiformis* increased the proliferation of T and B lymphocytes, enhancing cellular and humoral immune responses [33]. In RAW264.7 cells, *G. lemaneiformis* upregulated iNOS, IL-6 and TNF-α genes, promoting cytokine production and pinocytosis [24]. In ovalbumin-induced allergic mice, *G. lemaneiformis* upregulated splenic CD4+ Foxp3+ T cell populations and increased serum IL-10 and TGF-β levels, indicating its anti-allergic activity [54].

Research on the neuroprotective effects of SPs from *G. cornea* showed reduced oxidative stress and monoamine content in 6-OHDA-induced Parkinson’s disease (PD) models in rats, suggesting their potential as a therapeutic candidate for PD [31]. In HT-22 cells, *G. gracilis* prevented zinc-induced damage by upregulating antioxidant enzymes, inhibiting apoptosis, oxidative damage and acetylcholinesterase activity, highlighting its neuroprotective potential in Alzheimer’s disease [13]. SPs from *G. fisheri* modulated gut microbiota composition and increased SCFA production in acetic acid-induced colitis models in ICR/Mlac mice, reducing colonic shortening and promoting gut smooth muscle contraction [51]. In Duroc × (Landrace × Yorkshire) pigs, *G. lemaneiformis* reduced harmful bacteria (e.g., *Shigella*) while increasing beneficial bacteria (e.g., *Clostridium* and *Lactobacillus*) [14]. In high-fat diet-fed BALB/c mice, *G. lemaneiformis* altered the gut microbiota and lipid gene expression via SCFAs, reducing hepatic and adipose fat accumulation [49]. In dextran sulphate sodium-induced colitis models in Kunming male mice, *G. lemaneiformis* inhibited CCL-25 and CCR-9 levels, increased CD40 and TGF-β1 and modulated the gut microbiota, suggesting its use as a functional food for ulcerative colitis patients [47].

## 6. Limitations and Future Prospects

Despite the promising therapeutic potential of *Gracilaria* SPs, several limitations need to be addressed. The bioactivity of SPs depends on the species, extraction/purification technique employed and oceanic parameters; this variability may affect their therapeutic applications. Most studies on *Gracilaria* SPs are preclinical and lack the support of clinical trials, leaving a significant gap in clinical evidence concerning their safety and effectiveness on human health. Although *Gracilaria* SPs are generally considered safe, potential drawbacks and long-term safety require further investigation.

The therapeutic potential of *Gracilaria* SPs is vast, but further research is needed to fully explore their applications. Future studies should focus on conducting well-designed clinical trials to evaluate the safety, efficacy, and optimal dosage of SPs in humans. Furthermore, molecular dynamics-related research is critical to understanding the specific signal transduction pathways through which *Gracilaria* SPs demonstrate their effects in managing chronic diseases. Developing advanced drug delivery systems, such as nanoparticles, could enhance the bioavailability and targeted delivery of SPs. Combining SPs with existing therapies may significantly improve treatment outcomes for chronic disease management. Furthermore, research should be focused on exploring the mechanisms of SPs in gut microbiota modulation and conducting clinical trials to evaluate their efficacy in treating gut-related disorders such as IBD (Inflammatory Bowel Disease) and IBS (irritable bowel syndrome). Synergistic effects with probiotics or prebiotics could enhance their therapeutic potential.

## 7. Conclusions

This review concludes that *Gracilaria* sulphated polysaccharides (SPs) exhibit significant therapeutic potential in managing chronic diseases through various mechanisms, including anticancer, anti-inflammatory, antidiabetic, immunomodulatory and neuroprotective activities. Animal model studies have demonstrated potent anticancer effects through various mechanisms, including apoptosis induction, immune cell modulation and signalling pathway inhibition, further enhanced by conjugating with nanobiotechnology. *Gracilaria* SPs also displayed anti-inflammatory properties by suppressing inflammatory mediators and modulating key inflammatory and complement pathways. Neuroprotective effects, including mitigation of oxidative stress and enhanced neuronal viability, suggest the potential for managing neurodegenerative diseases. In diabetes models, SPs improved glucose homeostasis and lipid metabolism, sometimes surpassing conventional treatments. Finally, their immune-modulatory activities highlighted their potential as functional food additives and immune-stimulating supplements. These findings highlighted SP interactions with multiple molecular pathways, offering a multifaceted approach to disease management. However, further research, particularly clinical trials and mechanistic studies, is essential to fully harness the therapeutic potential of SPs. With continued exploration, *Gracilaria* SPs could pave the way for novel treatments targeting chronic diseases, ultimately improving human health and well-being.

## Data Availability

All data are provided in the article.
